# Predictive model for postoperative hyperalgesia in patients with lower limb fractures undergoing nerve block surgery

**DOI:** 10.12669/pjms.42.5.14933

**Published:** 2026-05

**Authors:** Pingping Pang, Hengyi Xu, Wei Du, Fengmei Dong

**Affiliations:** 1 Pingping Pang Department of Anesthesiology, Huzhou Nanxun Hospital of Traditional Chinese Medicine, Huzhou, Zhejiang Province 313009, P.R. China; 2 Hengyi Xu Department of Orthopedics, Huzhou Nanxun Hospital of Traditional Chinese Medicine, Huzhou, Zhejiang Province 313009, P.R. China; 3 Wei Du Department of Anesthesiology,Huzhou Central Hospital, Huzhou, Zhejiang Province 313000, P.R. China; 4 Fengmei Dong Operating Room, Huzhou Nanxun Hospital of Traditional Chinese Medicine, Huzhou, Zhejiang Province 313009, P.R. China

**Keywords:** Hyperalgesia, Lower limb fracture, Multifactor analysis, Nerve block, Nomogram

## Abstract

**Objective:**

To establish and validate a predictive model for postoperative hyperalgesia in patients with lower limb fractures undergoing nerve block surgery.

**Methodology:**

This retrospective study analyzed 168 patients receiving postoperative nerve block analgesia at Huzhou Nanxun Hospital of Traditional Chinese Medicine (January 2022-October 2024). Patients were stratified into hyperalgesia and non-hyperalgesia groups. Logistic regression identified independent risk factors, followed by the development and validation of a nomogram model using R.

**Results:**

Hyperalgesia incidence was 14.88% (25/168). Significant differences existed in smoking history, diabetes history, operation time, distal compression at the injection site at injection site, and ropivacaine dosage (P<0.05). Multivariate analysis confirmed diabetes history (OR=4.12), operation time (OR=3.85), distal compression at the injection site (OR=0.24), and ropivacaine dosage (OR=3.21) as independent predictors (P<0.05). The nomogram achieved an AUC of 0.819 (95%CI: 0.732-0.906), with a calibration curve slope of 1 and an intercept <0.001. Decision curve analysis showed optimal net benefit at threshold probabilities between 0.05 and 0.55.

**Conclusion:**

Postoperative hyperalgesia is primarily associated with a history of diabetes, prolonged operation time, absence of distal compression at the injection site, and higher ropivacaine dosage. The validated nomogram provides accurate individualized risk prediction.

## INTRODUCTION

Lower limb fractures are one of the most common types of trauma in clinical practice. Survey data show that there are over 10 million patients with lower limb fractures in China each year, primarily involving fractures of the femur, tibia, and fibula. Surgery is the main treatment method.^1^ Postoperative pain management for lower limb fractures directly affects patients’ early mobility, rehabilitation progress, and incidence of complications. In recent years, nerve block techniques have become an important means of postoperative analgesia for lower limb fractures due to their precise analgesic effects, significant reduction in opioid usage, and few systemic adverse reactions.^2^ However, with the widespread adoption of this technique, postoperative hyperalgesia has gradually become more prominent: some patients experience an abnormal decrease in pain threshold, an expanded pain range, or severe pain responses to non-nociceptive stimuli (such as temperature and touch) after the nerve block wears off.^3^,^4^ This state of hyperalgesia not only severely reduces patients’ quality of life but may also lead to chronic pain, limb dysfunction, and even psychological issues such as anxiety and depression, significantly prolonging hospital stays and increasing medical costs.

The mechanism of hyperalgesia is complex, involving multiple aspects such as central sensitization, peripheral nerve injury, and inflammatory response.^5^-^7^ Factors such as surgery duration and local anesthetic dose may be associated with it. In clinical practice, if high-risk patients for hyperalgesia can be identified early, the incidence can be effectively reduced by adjusting the anesthesia plan (such as optimizing the dosage of local anesthetics, combining multimodal analgesia) or intervening in advance (such as prophylactic use of anticonvulsants). However, due to the extensive trauma and complex surgical procedures (such as intramedullary nail fixation and joint replacement) involved in lower limb fractures, the influencing factors of postoperative hyperalgesia may differ from those in fractures of other locations. Currently, systematic research on hyperalgesia after nerve block in patients with lower limb fractures is still lacking. In addition, there is a lack of simple, quantitative prediction tools in clinical practice, and anesthesiologists often rely on empirical judgment, which can easily lead to missed diagnoses in high-risk patients or excessive interventions in low-risk patients. As a visual risk prediction model, a nomogram can integrate multiple factors and quantify individual risks and has been widely used in fields such as tumor prognosis and cardiovascular events. In view of this, this study aimed to establish a predictive model for hyperalgesia after nerve block in patients with lower-limb fractures, providing new insights into its early diagnosis and treatment.

## METHODOLOGY

A retrospective analysis was conducted on 168 patients with lower limb fractures who underwent postoperative analgesia via nerve block at Huzhou Nanxun Hospital of Traditional Chinese Medicine between January 2022 to October 2024.

### Ethical Approval:

This study was reviewed and approved by the hospital ethics committee (approval number: 2025-L-02-01; dated February 1, 2025).

### Inclusion criteria:

- Lower limb fracture confirmed by imaging.
- Age ≥ 18 years.
- Pain sensitivity assessment conducted within 24 hours postoperatively.
- Unilateral involvement.
- Complete relevant data available for analysis.

### Exclusion criteria:

- Patients with open fractures.
- Patients with concomitant infectious diseases.
- Patients with malignant tumors.

### Nerve block procedure:

In this study, nerve blocks administered to patients with lower-limb fractures were the core component of postoperative multimodal analgesia. Depending on the fracture location and surgical site, the primary targets for ultrasound-guided nerve blocks were selected to anesthetize sensory nerves innervating the affected limb: such as Femoral Nerve Block (FNB): Applicable for fractures involving the anterior thigh, knee joint, and parts of the lower leg (e.g., femur, patella fractures); Sciatic Nerve Block (SNB): Applicable for fractures involving the posterior thigh, posterior knee, lower leg, and ankle/foot region (e.g., tibia/fibula, ankle fractures). The specific procedural workflow for nerve blocks was as follows: Patients were placed in a comfortable position (supine or lateral decubitus) to fully expose the target area. A B-scan ultrasound high-frequency linear array probe (13-6 MHz) was used. Puncture was performed using either the in-plane or out-of-plane technique. The needle tip was adjusted near the sheath of the target nerve. After negative aspiration confirmed no blood return, the local anesthetic (0.5% ropivacaine) was injected slowly. Based on patient weight, body habitus, and the required extent of block coverage, the ropivacaine dose was individualized, with adequate volume maintained to achieve an effective blockade. A dose of ropivacaine of less than 1.5 mg/kg was considered a low dose, and 1.5–3 mg/kg was considered a high dose.

### Data collection:

The following indicators were collected from all patients:

### General details:

gender, age, smoking history, alcohol consumption history, history of hypertension, history of diabetes mellitus, history of hyperlipidemia;

### Surgical-related indicators:

The American Society of Anesthesiologists (ASA) physical status classification, duration of surgery, intraoperative blood loss, surgical technique, fracture location, affected limb, distal compression at the injection site, ropivacaine dosage administered, and intraoperative bone grafting.

### Assessment of hyperalgesia:

Pain levels were evaluated using the Visual Analogue Scale (VAS) within 24 hours post-surgery. This scale ranges from 0 to 10, with higher scores indicating more pronounced pain. Based on VAS results, hyperalgesia was diagnosed if two or more criteria were met:^8^ 1. Expansion of the painful area; 2. Persistent and intense pain at the site of injury; 3. Pain induced by thermal stimuli (e.g., heat, cold); 4. Sustained VAS score exceeding 8 points; 5. Requirement for increased analgesic dosage postoperatively; 6. Presence of psychological symptoms or protective limb movements.

### Model development and validation:

Patients were assigned to hyperalgesia and non-hyperalgesia groups based on the presence or absence of hyperalgesia. General characteristics and surgery-related indicators were compared between the groups. Logistic regression was used to assess independent factors associated with hyperalgesia following nerve block in patients with lower limb fractures. A stepwise logistic regression model was constructed using R^9^ and validated using receiver operating characteristic (ROC) and calibration curves. Clinical applicability was evaluated through decision curve analysis. Due to sample size constraints, this study did not separate the data into training and validation sets. Internal validation was performed using repeated sampling. The completed Transparent Reporting of a multivariable prediction model for Individual Prognosis Or Diagnosis (TRIPOD) checklist is provided as a Supplementary file.

**Supplementary Table:** TRIPOD Checklist: Prediction Model Development and Validation

Section/Topic	Item		Checklist Item	Page
***Title and abstract***				
Title	1	D;V	Identify the study as developing and/or validating a multivariable prediction model, the target population, and the outcome to be predicted.	1
Abstract	2	D;V	Provide a summary of objectives, study design, setting, participants, sample size, predictors, outcome, statistical analysis, results, and conclusions.	2
***Introduction***				
Background and objectives	3a	D;V	Explain the medical context (including whether diagnostic or prognostic) and rationale for developing or validating the multivariable prediction model, including references to existing models.	3
	3b	D;V	Specify the objectives, including whether the study describes the development or validation of the model or both.	3
Methods				
Source of data	4a	D;V	Describe the study design or source of data (e.g., randomized trial, cohort, or registry data), separately for the development and validation data sets, if applicable.	3
	4b	D;V	Specify the key study dates, including start of accrual; end of accrual; and, if applicable, end of follow-up.	4
Participants	5a	D;V	Specify key elements of the study setting (e.g., primary care, secondary care, general population) including number and location of centres.	4
	5b	D;V	Describe eligibility criteria for participants.	4
	5c	D;V	Give details of treatments received, if relevant.	4
Outcome	6a	D;V	Clearly define the outcome that is predicted by the prediction model, including how and when assessed.	4
	6b	D;V	Report any actions to blind assessment of the outcome to be predicted.	4
Predictors	7a	D;V	Clearly define all predictors used in developing or validating the multivariable prediction model, including how and when they were measured.	4
	7b	D;V	Report any actions to blind assessment of predictors for the outcome and other predictors.	4
Sample size	8	D;V	Explain how the study size was arrived at.	4
Missing data	9	D;V	Describe how missing data were handled (e.g., complete-case analysis, single imputation, multiple imputation) with details of any imputation method.	4
Statistical analysis methods	10a	D	Describe how predictors were handled in the analyses.	4
	10b	D	Specify type of model, all model-building procedures (including any predictor selection), and method for internal validation.	4
	10c	V	For validation, describe how the predictions were calculated.	4
	10d	D;V	Specify all measures used to assess model performance and, if relevant, to compare multiple models.	4
	10e	V	Describe any model updating (e.g., recalibration) arising from the validation, if done.	4
Risk groups	11	D;V	Provide details on how risk groups were created, if done.	4
Development vs. validation	12	V	For validation, identify any differences from the development data in setting, eligibility criteria, outcome, and predictors.	5
***Results***				
Participants	13a	D;V	Describe the flow of participants through the study, including the number of participants with and without the outcome and, if applicable, a summary of the follow-up time. A diagram may be helpful.	5
	13b	D;V	Describe the characteristics of the participants (basic demographics, clinical features, available predictors), including the number of participants with missing data for predictors and outcome.	5
	13c	V	For validation, show a comparison with the development data of the distribution of important variables (demographics, predictors and outcome).	5
Model development	14a	D	Specify the number of participants and outcome events in each analysis.	5
	14b	D	If done, report the unadjusted association between each candidate predictor and outcome.	5
Model specification	15a	D	Present the full prediction model to allow predictions for individuals (i.e., all regression coefficients, and model intercept or baseline survival at a given time point).	5
	15b	D	Explain how to the use the prediction model.	5
Model performance	16	D;V	Report performance measures (with CIs) for the prediction model.	5
Model-updating	17	V	If done, report the results from any model updating (i.e., model specification, model performance).	5
Discussion				
Limitations	18	D;V	Discuss any limitations of the study (such as nonrepresentative sample, few events per predictor, missing data).	6
Interpretation	19a	V	For validation, discuss the results with reference to performance in the development data, and any other validation data.	7
	19b	D;V	Give an overall interpretation of the results, considering objectives, limitations, results from similar studies, and other relevant evidence.	8
Implications	20	D;V	Discuss the potential clinical use of the model and implications for future research.	8
Other information				
Supplementary information	21	D;V	Provide information about the availability of supplementary resources, such as study protocol, Web calculator, and data sets.	/
Funding	22	D;V	Give the source of funding and the role of the funders for the present study.	/

*Items relevant only to the development of a prediction model are denoted by D, items relating solely to a validation of a prediction model are denoted by V, and items relating to both are denoted D;V. We recommend using the TRIPOD Checklist in conjunction with the TRIPOD Explanation and Elaboration document.

### Statistical analysis:

Data was analyzed using R version 4.2.3. (R Development Core Team, Vienna, Austria**)** The count data were analyzed using χ² tests, and the continuous data were assessed using t-tests. Independent predictors were identified via multivariate logistic regression to construct a risk prediction model, which was visualized as a column chart. Model discrimination was assessed using ROC curves, while model accuracy was evaluated through calibration curves and H-L goodness-of-fit tests. Clinical efficacy was analyzed using decision curves. All analyses used P < 0.05 as the threshold for statistical significance.

## RESULTS

Clinical data of 183 patients with lower limb fractures who underwent postoperative analgesia via nerve block. Of them, nine patients with open fractures, four patients with concurrent infectious diseases, and two patients with malignant tumors were excluded. The final cohort comprised 168 patients (82 males and 86 females), aged 21 to 73 years, with a mean age of 34.93 ± 7.05 years. Of 168 patients, 25 (14.88%) developed hyperalgesia. Statistically significant differences were observed between the hyperalgesia and non-hyperalgesia groups for smoking history, history of diabetes, duration of surgery, distal compression at the injection site, and ropivacaine dosage (P < 0.05), as shown in Table-I. Incorporating the identified significant factors into a multivariate logistic regression analysis revealed that a history of diabetes, duration of surgery, distal compression at the injection site at the injection site, and ropivacaine dosage were independent factors influencing postoperative hyperalgesia in patients with lower limb fractures (P < 0.05), as shown in Table-II.

**Table I:** Comparison of baseline data between the hyperalgesia group and the non-hyperalgesia group.

Factors		Hyperalgesia Group (n=25)	Non-Hyperalgesia Group (n=143)	χ^2^/t	P
Gender	Male	13	69	0.120	0.729
	Female	12	74		
Age (year)		35.02±7.11	34.91±6.82	0.074	0.941
Smoking	No	12	111	9.522	0.002
	Yes	13	32		
Alcohol Consumption	No	18	105	0.022	0.882
	Yes	7	38		
Hypertension	No	20	121	0.336	0.562
	Yes	5	22		
Diabetes	No	19	131	5.419	0.020
	Yes	6	12		
hyperlipidemia	No	21	122	0.029	0.865
	Yes	4	21		
ASA stage	I~II	20	126	1.230	0.267
	III	5	17		
Surgery time	<60min	15	122	9.063	0.003
	≥60min	10	21		
Intraoperative blood loss (ml)		271.82±37.04	285.13±42.11	-1.483	0.140
Surgical procedure	Internal fixation	22	136	1.919	0.166
	External fixation	3	7		
Site of fracture	Femur	6	35	1.981	0.576
	Tibia and fibula	10	73		
	Patella	7	24		
	Calcaneus	2	11		
Side distinction	Left side	11	89	0.978	0.323
	Right side	14	74		
Distal compression at the injection site at the injection site	No	20	71	7.895	0.005
	Yes	5	72		
Ropivacaine usage	Low dose	17	51	9.235	0.002
	High dose	8	92		
Intraoperative bone grafting	No	17	95	0.023	0.878
	Yes	8	48		

**Table-II:** Multifactorial analysis of Hyperalgesia following nerve blockade in patients with Lower Limb Fractures.

*Factor*	*B*	*SE*	*Wald*	*P*	*OR*	*OR (95% CI)*	
						*Lower limit*	*Upper limit*
Smoking	0.913	0.487	3.515	0.061	2.492	0.959	6.472
Diabetes	1.442	0.645	5.005	0.025	4.230	1.196	14.964
Surgery time	1.899	0.578	10.783	0.001	6.677	2.150	20.739
Distal compression at the injection site at the injection site	-1.960	0.636	9.483	0.002	0.141	0.040	0.490
Ropivacaine usage	-1.382	0.510	7.338	0.007	0.251	0.092	0.682
Constant	-1.166	0.374	9.733	0.002	0.312		

**Fig.1:**
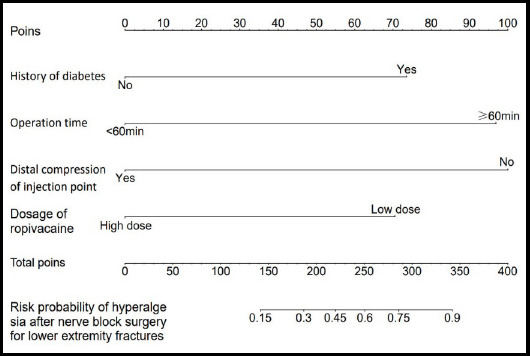
Prediction model for postoperative hyperalgesia following nerve blockade in patients with lower limb fractures.

**Fig.2:**
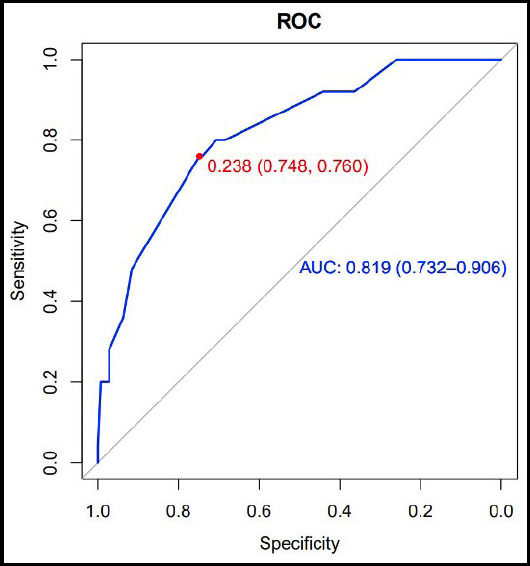
ROC Curve.

**Fig.3:**
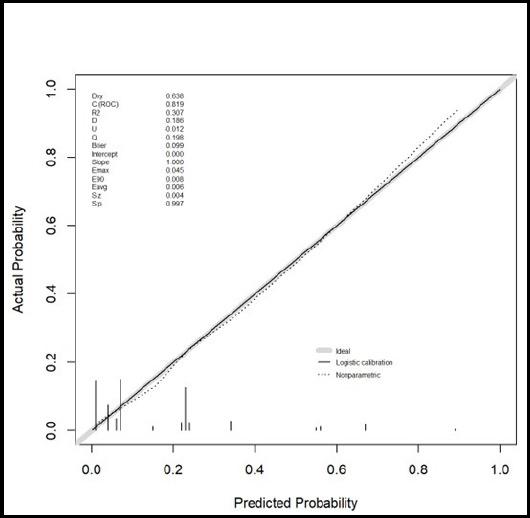
Calibration curve.

**Fig.4:**
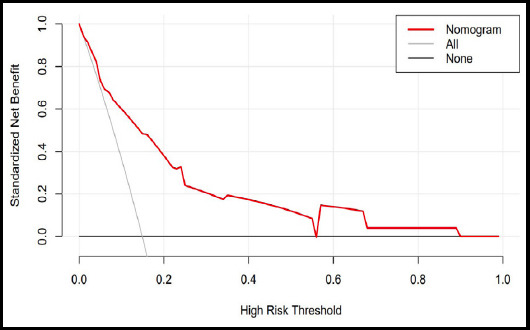
Decision curve.

Using R software, the results of the multivariate logistic regression analysis were visualized as a regression plot to establish a predictive regression model for postoperative hyperalgesia following nerve block in patients with lower limb fractures (Fig.1). Area under the ROC curve (AUC) of the model was 0.819, with a 95% confidence interval (CI) of (0.732-0.906), demonstrating good discriminating ability, as shown in Fig.2. The calibration curve slope was 1, with an intercept <0.001, and the model approximated the ideal diagonal line, confirming its accuracy (Fig.3). Clinical effectiveness analysis indicated that the net benefit of using this model to predict post-nerve-block hyperalgesia in lower-limb fracture patients was highest when the predictive probability threshold ranged from 0.05 to 0.55, as depicted in Fig.4.

## DISCUSSIONS

This study showed that the history of diabetes, operation time, distal compression at the injection site at the injection point, and other factors were associated with the development of hyperalgesia in patients with lower limb fractures after nerve block surgery. The nomogram model developed from the identified risk factors has high accuracy and discriminative power in predicting hyperalgesia.

While clinical treatment of lower limb fractures may include both surgical procedures and non-surgical methods, surgical intervention remains the mainstay of clinical treatment for severe cases.^10^ While postoperative analgesia using nerve block can effectively reduce the risk of complications such as somnolence, bronchospasm, and delirium in patients undergoing lower limb fracture surgery,^11^ some patients may develop a postoperative hyperalgesia, an exaggerated response to painful stimuli after the lower limb surgery.^7^

Among the 168 patients included in this study, 25 (14.88%) experienced hyperalgesia, a finding comparable to the study by Lin Qinying et al. on hyperalgesia following brachial plexus nerve block in patients with radial fractures.^12^

The nomogram model employed in this study integrates various influencing factors into a unified, visually predictive model via line segments, enabling personalized, visualized, and quantified prediction of hyperalgesia risk in these patients. This model has been applied to predict multiple clinical events.^13^ The line graph model offers high visualization, straightforward interpretation, and ease of comprehension. For populations at high risk of postoperative hyperalgesia, targeted intervention measures can be implemented. This approach not only enhances the precision of interventions but also conserves healthcare resources by sparing low-risk individuals from unnecessary interventions, thereby reducing medical expenditure.

Univariate and multivariate logistic regression analyses in this study identified a history of diabetes, duration of surgery, distal compression at the injection site at the injection site, and ropivacaine dosage as independent factors influencing postoperative hyperalgesia in patients with lower limb fractures (P<0.05). The impact of a history of diabetes on postoperative hyperalgesia in patients with lower limb fractures may be related to the hyperglycaemic state in diabetic patients, which can lead to metabolic disorders in nerve fibers, microvascular lesions, and inflammatory responses, subsequently triggering nerve damage and abnormal pain conduction.^14^ Moreover, diabetic patients exhibit impaired nerve repair capacity, rendering them susceptible to chronic pain and hyperalgesia following nerve injury.^15^ Therefore, while nerve block temporarily alleviates pain by interrupting pain signal transmission, the subsequent withdrawal of the block may trigger abnormal amplification of pain signals due to underlying nerve damage and inflammatory responses in diabetic patients. This manifests as ‘rebound pain’ or ‘flare-up pain’.^16^ In such patients, multimodal analgesia that combines nerve blocks, non-steroidal anti-inflammatory drugs (NSAIDs), opioids, and anticonvulsants (e.g., pregabalin) may be more effective in alleviating postoperative pain and hyperalgesia.

Numerous studies have confirmed this study’s observation that surgical duration, as a significant perioperative variable, may influence postoperative hyperalgesia in patients with lower limb fractures.^17^,^18^ Prolonged surgery may increase the release of pro-inflammatory factors due to expanded tissue trauma and exacerbated ischemia-reperfusion injury, thereby activating peripheral and central sensitization mechanisms and inducing hyperalgesia.^19^ During lower limb fracture surgery, peripheral nerves, such as the tibial nerve, may be directly injured by traction, compression, or surgical manipulation.^20^ Prolonged operative time increases nerve exposure to mechanical stress, causing perineural oedema and axonal damage, which further exacerbates postoperative neuropathic pain and hyperalgesia.^21^

Commonly used nerve block agents (e.g., ropivacaine, bupivacaine) typically provide analgesia for 4-6 hours. However, in cases of prolonged surgical time, the postoperative decline in nerve block effects may precede the peak inflammatory phase, creating an ‘analgesic gap’ that triggers rebound hyperalgesia.^21^,^22^ Therefore, for anticipated lengthy procedures, long-acting local anesthetics (e.g., 0.5% ropivacaine) or combination adjuvants (e.g., dexamethasone) may be more beneficial to prolong block duration. Additionally, employing multimodal analgesia (NSAIDs + nerve block + opioids) can reduce reliance on any single modality, thereby lowering the risk of hyperalgesia. Patients undergoing prolonged procedures may benefit from early prophylactic analgesia (e.g., gabapentin, pregabalin) postoperatively to inhibit central sensitization.

Additionally, distal compression at the injection site at the injection site during nerve block may facilitate the diffusion of local anesthetics within the nerve sheath through mechanical action.^23^ For instance, in sciatic nerve block, the popliteal approach, due to abundant fat and connective tissue within the nerve sheath, was shown to restrict local anesthetic diffusion, resulting in prolonged onset times.^24^ distal compression at the injection site may improve drug distribution, shorten onset times, and enhance block efficacy, thereby reducing postoperative pain or hyperalgesia caused by incomplete blockage.^25^ Inadequate ropivacaine dosage may compromise anesthetic depth, thereby diminishing pain conduction inhibition and central sensitization suppression, increasing the risk of hyperalgesia. Clinicians should therefore closely monitor anesthetic depth to mitigate this risk.^26^

Validation of the nomogram model, developed in this study, demonstrated an AUC > 0.7, indicating that the model possesses excellent discriminatory capability and can accurately identify high-risk individuals for postoperative hyperalgesia among patients with lower limb fractures. Calibration curves confirmed that the model’s predicted high-risk cohort closely approximated the actual incidence of hyperalgesia, reflecting high predictive accuracy. These results have clear clinical significance. AUC values for predictive models of perioperative hyperalgesia typically range from 0.75 to 0.85.^27^,^28^ The AUC of the present model (0.819) lies at the higher end of this range, and the calibration curve (slope = 1, intercept < 0.001) demonstrates close agreement between predicted probabilities and observed outcomes, indicating good model stability. However, compared with some models developed for chronic or neuropathic pain, which show AUCs> 0.85 (REF), the model’s performance may be further improved. The model’s limitations may be attributable to the use of single-center retrospective data, the relatively small sample size (168 cases), and the homogeneity of the study cohort, all of which may have constrained its performance.

### Strength of the study:

A key strength of this report is that it is one of the few studies specifically focused on investigating postoperative hyperalgesia in patients with lower-limb fractures undergoing nerve block. The developed model integrates multiple clinical variables, such as diabetes history, duration of surgery, and distal compression at the injection site, into a nomogram, providing a user-friendly, personalized risk assessment tool that can be readily applied in clinical settings. Additionally, rigorous statistical validation (AUC=0.819, calibration slope=1) underscores the model’s robust discriminatory power and accuracy, bolstering its clinical utility in identifying high-risk patients. Finally, detailed descriptions of nerve block techniques (such as ultrasound guidance, ropivacaine dose titration) and diagnostic criteria for hyperalgesia (based on visual analog scale and clinical symptoms) ensure methodological consistency and reproducibility.

### Limitations:

Firstly, this is a single-center retrospective study with a limited sample size. Subsequent multicenter, large-sample prospective intervention trials are needed to verify whether the risk-stratified analgesia regimen based on nomograms effectively reduces the incidence of hyperalgesia, thereby further evaluating the model’s clinical value. Secondly, external validation is needed in multicenter cohorts with diverse patient populations (e.g., age groups, fracture types) to confirm the predictive performance of nomograms across different clinical settings. In addition, long-term follow-up studies are needed to evaluate the model’s predictive ability for chronic hyperalgesia or persistent pain after the acute phase. Finally, future studies should explore the molecular mechanisms underlying the identified risk factors, such as diabetes-related neuroinflammation, to provide a theoretical basis for developing targeted prevention strategies.

## CONCLUSIONS

Postoperative hyperalgesia following nerve block in patients with lower limb fractures is primarily influenced by factors such as a history of diabetes, duration of surgery, and distal compression at the injection site at the injection site. The nomogram model developed based on these factors demonstrates high accuracy and discriminatory power in predicting postoperative hyperalgesia following nerve block in patients with lower limb fractures.

### Authors’ contributions:

**PP and FD:** Literature search, study design and manuscript writing, revision and validation and is responsible for the integrity of the study.

**WD and HX:** Data collection, data analysis, interpretation and critical review.

All authors have read and approved the final manuscript.
